# Sleep Stage Monitoring in Congenital Heart Disease (CHD) Using a Digital Health Application Programming Interface (API)

**DOI:** 10.3390/jcm14228097

**Published:** 2025-11-15

**Authors:** Charlotte Schöneburg, Isabel Uphoff, Viktoria Ludwig, Renate Oberhoffer-Fritz, Peter Ewert, Jan Müller

**Affiliations:** 1 Institute of Preventive Pediatrics, TUM School of Medicine and Health, Technische Universität München, Am Olympiacampus 11, 80809 München, Germany; 2 Department of Congenital Heart Disease and Pediatric Cardiology, TUM Klinikum Deutsches Herzzentrum, Lazarettstrasse 36, 80636 München, Germany

**Keywords:** congenital heart disease, health application programming interface, telemedicine, sleep

## Abstract

**Background**: Adults with congenital heart disease (CHD) are living longer but face increasing comorbidities. Sleep is a key health determinant, yet objective data in CHD remain limited. This study compared sleep characteristics of adults with CHD and controls using wearable technology and a Health Application Programming Interface (API). **Methods**: A total of 175 CHD patients (33.1 ± 10.3 years, 49.2% women) and 52 controls (34.4 ± 12.4 years, 40.4% women) completed seven continuous days of wrist-worn Garmin Vivosmart^®^ 5 during routine follow-up at the TUM Klinikum Deutsches Herzzentrum. Sleep duration, phases, Sleep Scores, and weekday-weekend differences were analyzed, and multivariate models examined clinical and demographic predictors. **Results**: Total sleep duration and rapid eye movement (REM) sleep did not differ between groups. CHD patients had more deep sleep (83 ± 19 vs. 75 ± 16 min, *p* = 0.004) but lower Sleep Scores (74 ± 9 vs. 77 ± 9, *p* = 0.041). Within CHDs, deep sleep was higher on weekends than on weekdays (*p* = 0.033). Multivariate analyses showed no overall group effect, but age (*p* = 0.016), sex (*p* = 0.013), and body mass index (BMI; *p* < 0.001) significantly predicted sleep outcomes. Regression analyses in CHDs revealed female sex associated with longer REM sleep (*p* < 0.001), while higher BMI consistently predicted poorer outcomes. Disease severity was linked to lower Sleep Scores. **Conclusions**: Sleep in CHDs is broadly comparable to controls, but BMI, sex, and disease severity significantly shape outcomes. The additional variability between weekends and weekdays and a higher risk of sleep-disordered breathing, according to the literature, underscores that sleep is an underestimated target for prevention and clinical care in CHD.

## 1. Introduction

Improvements in medical and surgical care have significantly increased the survival rates and life expectancies of people living with congenital heart disease (CHD) across all age groups [1,2]. Next to managing the congenital condition itself, the healthcare system is thus faced with age-related comorbidities. In order to improve long-term health outcomes in this growing patient population, it is essential to understand the interplay between CHD and these secondary health issues. Digital health platforms or application programming interfaces (API) have built a new-emerging opportunity for real-world data acquisition, which enables the objective assessment of lifestyle factors, such as sleep, that are often challenging to evaluate via self-report. A systematic review summarized recent policy-driven initiatives and further highlights that approaches such as telemedicine, mobile health tools, wearable monitoring, and artificial intelligence systems are increasingly used to improve access to care and long-term disease management [3].

Sleep is a critical factor influencing overall health. Poor or insufficient sleep has been linked, among others, to overall mortality, cardiovascular disease, accidents and injuries, and diabetes [4]. In addition, inadequate sleep can contribute to obesity, cognitive impairments, and other adverse health outcomes [5]. The optimal sleep duration, according to the National Sleep Foundation, is 7 to 9 h per night [6]. Both short (≤5 h) but also long (≥10 h) sleep durations were associated with increased risks of stroke and coronary heart disease [7]. While sleep duration is an important component, it represents only one part of the concept of sleep. Other aspects, such as sleep depth, quality, and phases, also play critical roles in determining overall sleep health [8]. Sleep is a vital factor for maintaining health and well-being as it affects cognitive function, physiological regulation, emotional stability, physical development, and overall quality of life [6].

Beyond total sleep time, differences in sleep phases (sleep architecture) have direct clinical implications: lower slow-wave sleep or deep sleep (N3) and fragmented rapid-eye movement (REM) are associated with autonomic imbalance, non-dipping blood pressure, arrhythmia susceptibility, metabolic dysregulation, and cognitive impairment, making stage-specific alterations clinically meaningful [9,10,11]. Altered nocturnal autonomic profiles seen in adults with CHD may reflect enduring effects of congenital and genetic factors shaped early in development. Structural and chromosomal abnormalities associated with CHD can disrupt cardiac conduction and autonomic balance, increasing vulnerability to rhythm disturbances and sudden cardiac death in childhood [12]. Even when surgically corrected, complex or syndromic heart defects identified prenatally often remain linked to less favorable long-term outcomes [13], which could partly account for the persistent differences in sleep-related autonomic regulation observed in adulthood.

In individuals with CHD, the consequences of sleep disturbances may be particularly important, as this group may have an increased risk for developing sleep-disordered breathing (SDB) [14]. Research on sleep in infants with CHD has revealed frequent episodes of oxygen desaturation during sleep [15], while studies in older children also indicate a higher prevalence of sleep-related difficulties compared to healthy peers [16]. Early identification and management of sleep issues in this population are therefore even more critical to support optimal neurodevelopmental outcomes and to prevent additional health complications.

Given this context, the present study aims to investigate two distinct aims. First, we compare sleep architecture, comprising sleep duration, stage distribution, and efficiency, including weekday–weekend differences, between adults with CHD and controls using wearable technology and a health API over seven consecutive days and nights. Second, we examine associations between sleep metrics and pre-specified patient characteristics within the CHD group. Candidate predictors are peak oxygen uptake ($\dot{V}O_{2}$ peak), body mass index (BMI), sex, CHD severity, and the number of prior cardiac surgeries. $\dot{V}O_{2}$ peak is a valuable prognostic marker in this patient group, as it is associated with clinical outcomes such as increased risk of hospitalization and all-cause mortality [17,18]. These exploratory models are hypothesis-generating and intended to inform future confirmatory work. 

## 2. Participants and Methods

### 2.1. Study Participants

A total of 219 adults aged 18 years and older with various forms of CHD, next to 54 heart-healthy controls, were enrolled between September 2023 to July 2025. Recruitment took place during their routine Cardio Pulmonary Exercise Testing (CPET) at the outpatient department of the TUM Klinikum Deutsches Herzzentrum. The control group consisted of adults with no history of cardiovascular or other chronic medical conditions. Participants were recruited from hospital staff, colleagues, and acquaintances and were not patients of the cardiology department. All control participants underwent the same study procedures and measurements as the CHD group and were tested following the same daytime protocol after the regular clinical schedule. Exclusion occurred due to technical difficulties, outliers, or missing data. After that, 175 adults (33.1 ± 10.3 years, 49.2% women) with CHD and 52 controls (34.4 ± 12.4 years, 40.4% women) completed a seven-day sleep tracking using wearable devices. All participants provided written informed consent after receiving an explanation of the study procedure. The study was carried out following the Declaration of Helsinki (2008 revision) and received approval from the ethics committee of the Technical University of Munich (2023-184-S-KK: approval date: 18 April 2023). It was registered as a cross-sectional study in the German Trials Registry (DRKS00032675: approval date: 20 September 2023).

Heart defect categories were classified according to the American College of Cardiology (ACC) and were critical for inclusion. It was distributed as 77 complex, 81 moderate, and 17 simple [19,20]. Cognitive handicaps, medication intake of chronotropic, dromotropic, and/or bathmotropic effects, cardiomyopathy, and pacemakers were exclusion criteria.

Within the scope of this project, patient characteristics such as anthropometry, age, and sex, as well as CPET parameters like the $\dot{V}O_{2}$ peak, which represents the highest mean oxygen consumption during exercise in mL/min/kg, were measured. Table 1 describes the sample characteristics.

### 2.2. Sleep Tracking

The Garmin Vivosmart^®^ 5 device (Garmin Ltd., Olathe, KS, USA), equipped with an integrated optical heart sensor (Garmin Elevate™), was used to measure sleep duration and phases over seven consecutive days. Monitored sleep phases, based on the common interpretation [21], were classified as light (N1, N2), deep (N3), REM, and awake. Based on these factors, in addition to restlessness, activation of the autonomic nervous system, and total sleep duration, an individual Sleep Score was calculated for each night for each participant. Sleep is rated on a scale of 0–100, which summarizes the overall quality of your sleep: Very good: 90–100, good: 80–89, adequate: 60–79, and poor: <60 [22]. A minimum of three nights was determined in order to obtain a valid estimate of average sleep duration [23]. Across the whole sample, 66.7% of participants had valid sleep data for all seven nights, while 20.8% had six nights, and 12.5% had between three and five nights. The wrist-worn device was linked to the ©Digital Rebels GmbH (Fitrockr Research & Analytics, Berlin, Germany) Health API through a smartphone application, which participants accessed on-site at the clinic. This setup allowed continuous and automated data transfer to a clinic’s internal cloud infrastructure. Participants were instructed to synchronize their devices with the app on a daily basis and to follow their usual daily routines. The wearable was worn during all everyday activities, including sleep, work, physical activity, and even showering. The only exception was a brief period of recharging the battery during the daytime.

### 2.3. Cardio Pulmonary Exercise Testing (CPET)

Exercise capacity was assessed using a symptom-limited CPET on an upright, electronically braked cycle ergometer with a standardized ramp protocol [18]. After three minutes of rest and a short, unloaded warm-up, the workload was progressively increased (10–40 W/min) according to individual tolerance, targeting a total duration of 8–12 min. Participants maintained 60–80 rpm until volitional exhaustion. Throughout the test and recovery, breath-by-breath gas exchange, ventilation, and electrocardiogram were continuously recorded; oxygen saturation and blood pressure were measured at two-minute intervals. Peak oxygen uptake ($\dot{V}O_{2}$ peak, mL/min/kg) was defined as the highest 30-s average during exercise. Predicted values were adjusted for age, sex, weight, and height and expressed as percentage of predicted $\dot{V}O_{2}$ peak [24,25]. Only participants who reached a respiratory exchange ratio (RER) > 1.05 were included to confirm metabolic exhaustion [24,25,26,27]. This criterion resulted in a final subsample of 195 participants for analysis.

### 2.4. Statistical Analyses

All descriptive data are shown as mean ± standard deviation (SD). Potential outliers were detected based on the interquartile range method (IQR rule). Values lying below Q1 − 1.5·IQR or above Q3 + 1.5·IQR were excluded [28]. Differences between weekday and weekend sleep were tested via the *t*-test. Thereby, for example, “Friday” refers to the sleep period from Friday night to Saturday morning. To examine overall group differences in sleep architecture, multivariate analysis of variance (MANOVA) was conducted. This was followed by a multivariate analysis of covariance (MANCOVA) controlling for age, sex, BMI, and cardiorespiratory fitness ($\dot{V}O_{2}$ peak). Subsequently, to identify predictors of sleep characteristics within the CHD group, univariate linear regressions for each potential predictor for deep, REM, and Sleep Score were performed. All variables showing significant associations (*p* ≤ 0.05) were included in the final multivariate regression models. All data were analyzed using RStudio software (2022.07.1). Data visualization, including coefficient plots, was performed using the “ggplot2” package in RStudio.

## 3. Results

The statistics revealed no significant difference in sleep duration in the CHD group (461 ± 50 min) compared to controls (458 ± 46 min, *p* = 0.691). Regarding specific sleep phases, individuals with CHD spent an average of 92 ± 26 min in REM sleep, which is not significantly different from controls (93 ± 25 min, *p* = 0.740). Deep sleep was significantly higher in CHDs (83 ± 19 min) compared to controls (75 ± 16 min, *p* = 0.004). Light sleep was not significantly different between the two groups (CHD: 269 ± 40 min vs. controls: 276 ± 36 min, *p* = 0.218), as well as awakening time (CHD: 21 ± 14 min vs. controls: 21 ± 14 min, *p* = 0.946). Mean Sleep Score was 74 ± 9 for the CHD group and 77 ± 9 for controls (*p* = 0.041) and therefore significantly lower in CHDs.

**Figure 1:**
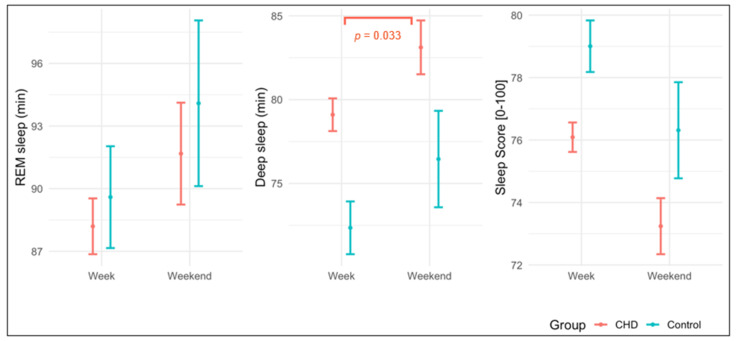
Differences in sleep parameters between weekdays and weekends. CHD, congenital heart disease; REM, Rapid Eye Movement; *p*-value significant with *p* < 0.05.

Within the CHD group and the controls, REM sleep did not significantly differ between weekdays and weekends (CHD: weekdays: 88 ± 37 min vs. weekends: 92 ± 44 min; *p* = 0.211; controls: weekdays: 90 ± 38 min vs. weekends: 94 ± 40 min; *p* = 0.336). For deep sleep, CHD patients exhibited significantly more deep sleep on weekends (83 ± 29 min) compared to weekdays (79 ± 27 min; *p* = 0.033). Among controls, these differences were not significant (weekdays: 72 ± 25 min vs. weekend: 76 ± 29 min; *p* = 0.213). The Sleep Score showed a trend difference between weekdays and weekends in CHDs (CHD: weekdays: 76 ± 13 vs. weekends: 73 ± 16; *p* = 0.088; controls: weekdays: 79 ± 13 vs. weekends: 76.3 ± 15.3; *p* = 0.210). Figure 1 describes the differences in sleep parameters between weekdays and weekends.

A one-way MANOVA, which considers all sleep phases as a combined cycle, found a trend in differences between the groups on the combined sleep phases (*p* = 0.081). Also, a MANCOVA controlling for age, sex, BMI, and $\dot{V}O_{2}$ peak supports the multivariate effect of the group (CHD vs. controls) on sleep phases (*p* = 0.083). Significant multivariate effects were observed for age (*p* = 0.016), sex (*p* = 0.013), and BMI (*p* < 0.001), indicating that older age, male sex, and higher BMI were linked to less favorable sleep outcomes.

Separate linear regression models were run to identify predictors of mean REM, deep sleep, and Sleep Score among the main target group: patients with CHD. Predictors for multivariate analysis were selected based on prior univariate results (Table 2).

In a reduced model that included sex, disease severity, BMI, and surgical history, both sex and BMI emerged as significant predictors of REM sleep duration (*p* < 0.001). Female sex was associated with significantly higher duration of REM sleep (*p* < 0.001), while higher BMI predicted substantially less REM sleep (*p* < 0.001). Disease severity and surgeries showed no significant effects. For deep sleep, BMI was retained as a trend-level predictor (*p* = 0.059).

In the reduced model for Sleep Score (*p* < 0.001), higher BMI was again a significant negative predictor (*p* = 0.001), which indicates that higher BMI predicts lower Sleep Score values. Compared to patients with complex CHD, both moderate (*p* = 0.017) and simple (*p* = 0.028) disease severity levels were associated with significantly higher Sleep Scores. Sex and surgical history were not significantly linked to Sleep Score.

## 4. Discussion

This study examined objective sleep characteristics in adults with CHD compared to control subjects. Unless otherwise specified, all analyses beyond the prespecified primary comparison are exploratory and hypothesis-generating; effect sizes and two-sided *p*-values are reported descriptively without adjustment for multiplicity and should be interpreted with appropriate caution. Total sleep duration and REM sleep did not differ, while CHD patients had more deep sleep but lower overall Sleep Scores. Overall, sleep architecture showed a trend but no significant multivariate group effect between groups. However, significant multivariate effects of age, sex, and BMI suggested that older age, male sex, and higher BMI were associated with less favorable sleep outcomes. Within the CHD group, sex and BMI proved to be important predictors: women had longer REM sleep, while a higher BMI was consistently associated with poorer outcomes. The severity of the disease was also relevant, with simple and moderate CHD associated with higher Sleep Scores than complex CHD. Comparisons between weekdays and weekends revealed significantly deeper sleep on weekends in CHD patients, suggesting a possible role for restorative sleep, while REM sleep and Sleep Scores remained nonsignificant.

This difference between weekends and weekdays is consistent with the general literature on sleep variability. In a large-scale study by the UK Biobank, sleep onset on weekends was, on average, 36 min later than on weekdays. Younger age, socioeconomic disadvantage, employment status, smoking status, male gender, being of non-white ethnicity, and later chronotype were found to predict greater deviations [29]. In contrast, among students, greater differences between weekdays and weekends were associated with poorer mental and somatic health and poorer academic performance. Importantly, among students who slept little during the week but extended their sleep on weekends, the negative effects were partially mitigated [30]. Evidence from the working population further supports this pattern: among nearly 2800 university employees, greater differences between weekday and weekend sleep duration were independently associated with stronger stress responses, while social jet lag (difference in the midpoint of the sleep phase) showed weaker associations [31]. Finally, brain activity during a visual attention task showed that longer sleep duration on weekends compared to weekdays was associated with better attention performance and healthier brain network function, while later sleep times on weekends were associated with poorer results [32]. Taken together, these studies show that discrepancies between weekday and weekend sleep are a robust phenomenon in the population and could impair both health, overall stress responses, and cognitive function.

Another relevant aspect of sleep health in CHD is the presence of SDB, such as obstructive sleep apnea (OSA). The results of this study, showing that a higher BMI predicts reduced REM sleep and lower Sleep Scores, are consistent with previous findings that OSA in adults with CHD is often closely related to increased BMI [33]. OSA is generally common among CHD patients, with approximately 31% of adults with CHD classified as being at high risk for OSA, which is a significantly higher prevalence than the 5 to 8% typically reported in the general population [33,34]. Beyond sleep disturbances, OSA is associated with a higher risk of diabetes, hypertension, and depression in CHD patients [33]. In pediatric CHD patients, OSA is even associated with adverse cardiac remodeling and reduced contractility [35]. In addition to weight, risk factors for OSA also include sex differences, with men twice as likely to suffer from OSA as women, and age, with an exponential increase in both middle-aged sexes [36]. The condition remains underdiagnosed in women, as they are more likely to experience symptoms such as mood swings, fatigue, morning headaches, nightmares, and insomnia than the typical male symptoms of snoring and shortness of breath [37]. These findings highlight that negative consequences of impaired sleep are pervasive in CHD, reinforcing the need for routine OSA screening and early intervention strategies.

Sleep is a multi-stage cycle that repeats around 4 to 6 times throughout the night [21]. In addition to quality and total duration, certain sleep phases also have special physiological and cognitive significance. Deep sleep (N3, slow-wave sleep) could be crucial for physical recovery, tissue repair, immune function, and long-term health, while REM sleep could be essential for regulating emotions, memory consolidation, and brain metabolism [21]. Both phases are often considered the most meaningful indicators of sleep quality [6,21]. It is therefore important to consider the most important sleep phases individually, but also to take into account the interaction of the sleep cycles and sleep as a whole. The higher proportion of deep sleep (N3) in adults with CHD should be interpreted cautiously and may reflect several potential mechanisms, including homeostatic rebound from accumulated sleep pressure, a heightened physiological recovery drive, or disease- and treatment-related effects (e.g., intermittent hypoxemia or cardiometabolic medication use) that alter sleep continuity. In addition, possible stage misclassification by consumer wearables cannot be excluded, underscoring the need for polysomnography-based, a diagnostic procedure recording brain, muscle, and cardiorespiratory activity during sleep, confirmation.

Most large-scale studies on sleep are still based on self-reported data, which is prone to memory bias and often lacks precision [7,38]. In this study, we used wearables linked to a health API in the participants’ everyday lives, which better reflects their actual sleep patterns. Patients can wear the wrist-worn wearable all night at home without any major disruption from light or the device itself, and then synchronize it with their smartphone in the morning. A health API provides a real, comprehensive, and immediate collection of raw data from individual patients. Even when compared to the gold standard for sleep recording, using polysomnography wearables can now record similarly decisive values. Limitations of studying sleep with wrist-worn trackers could be a marginal reduction in precision in distinguishing specific sleep stages or an overestimation of total sleep time [39,40,41,42]. In particular, there may be REM overestimation with underestimation of N3/waking state. In addition, there may be a reliance on PPG-derived HR/HRV, which is susceptible to artifacts, blood flow/skin color, and arrhythmias, as well as proprietary, evolving algorithms that compromise comparability [41,43].

Although previous validation studies have focused on earlier versions or similar Garmin Vivosmart models, these share comparable sensor technology. Wearables still offer a good compromise between feasibility and objectivity with acceptable sleep/wake discrimination [41,44,45].

Another limitation of the study is its cross-sectional design, which does not allow for interpretations of causal relationships but shows a snapshot of associations between key variables in a real-world setting. Furthermore, conclusions about long-term trends cannot necessarily be drawn from a 7-day observation period. Participants were not blinded to their device-generated Sleep Score on the wearable during the tracking week, which could have introduced expectancy effects/Hawthorne behavior (e.g., earlier bedtimes, reduced evening screen time) and response bias in self-reported covariates. However, the accompanying application did not display any detailed sleep information. While sleep was passively recorded without coaching or prompts, and the observation window was short, we cannot exclude reactivity to feedback. Future studies should blind participants to stage/Score outputs (or provide sham/minimal feedback) and include measures to quantify reactivity.

In summary, various risk factors, especially in CHD, such as BMI, severity of the disease, but also sex, influence the sleep phases. In addition, differences were apparent in CHD patients when comparing weekdays and weekends (deeper sleep on weekends in CHD patients). These results, in the context of the literature presented, emphasize the need to pay more attention to sleep as part of overall healthcare and individual health outcomes. Regular OSA screenings for men and women, targeted BMI management, and the promotion of stable sleep–wake rhythms should be carried out as part of holistic care. Lifestyle interventions can also play a role: physical activity is a recognized non-pharmacological strategy for improving sleep and is often recommended as an alternative or supplement to conventional therapies [44]. Cross-sectional data show that healthy physically active adolescents report better sleep quality than inactive peers [46], with moderate exercise appearing to be more beneficial than intense activity [47].

Future longitudinal studies are needed not only to examine these associations in greater depth but also to collect more comprehensive sleep data in particularly vulnerable patient groups. Such data could help identify actionable targets in real time, for example, through sleep coaching or tailored exercise programs aimed at prevention. The integration of health APIs with real-time analytics, supported by algorithms and deep learning, may further enable the early detection of critical health indicators and timely intervention.

## Figures and Tables

**Table 1:** Sample characteristics.

Variable	CHD	Control	*p*-Value *
Total (n)	175	52	
Sex (female) in n (%)	86 (49%)	21 (40%)	0.340
Age (Mean ± SD) in years	33.1 ± 10.3	34.4 ± 12.4	0.498
BMI (Mean ± SD) in kg/m^2^	24.3 ± 4.0	24.0 ± 3.7	0.575

CHD, Congenital Heart Disease; BMI, Body Mass Index; * comparing CHD with the controls, significant with *p* < 0.05.

**Table 2:** Summary of univariate and reduced multivariate linear regression models for REM sleep, deep sleep, and Sleep Score in the CHD sample.

Predictor	β REM Univ.	*p*-Value	β REM Multiv.	*p*-Value	β Deep Univ.	*p*-Value	β Deep Multiv.	*p*-Value	β Sleep Score Univ.	*p*-Value	β Sleep Score Multiv.	*p*-Value
Age (years)	−11.55	0.314	–	–	−3.7	0.657	–	–	0.03	0.569	–	–
Sex (female)	889.9	<0.001	797.3	<0.001	27.6	0.871	–	–	3.32	0.018	2.52	0.063
$\dot{V}O_{2}$ peak (mL/kg/mi)	5.07	0.418	–	–	0.1	0.977	–	–	0.07	0.058	–	–
Severity: moderate (complex)	537.4	0.029	309.1	0.216	−98.2	0.586	–	–	4.09	0.005	3.75	0.017
Severity: simple (complex)	925.4	0.025	516.4	0.254	40.2	0.894	–	–	7.07	0.004	6.27	0.028
BMI (kg/m^2^)	−109.1	<0.001	−104.1	<0.001	40.6	0.059	–	–	−0.56	0.002	−0.56	0.001
Surgeries: 1–2 (none)	−439.2	0.111	−243.2	0.410	323.7	0.108	–	–	−2.99	0.075	−0.98	0.595
Surgeries: 3+ (none)	−1064	0.005	−793.1	0.056	219.4	0.425	–	–	−4.54	0.047	−0.96	0.707

CHD, Congenital Heart Disease; BMI, Body Mass Index; REM, Rapid Eye Movement; $\dot{V}O_{2}$ peak, peak oxygen uptake; β as a regression coefficient for a predictor; *p*-value significant with *p* < 0.05.

## Data Availability

Datasets presented in this article are not readily available due to privacy and ethical restrictions. Requests to access the datasets should be directed to the corresponding author.
